# Soluble Fas ligand inhibits angiogenesis in rheumatoid arthritis

**DOI:** 10.1186/ar2181

**Published:** 2007-04-26

**Authors:** Wan-Uk Kim, Seung-Ki Kwok, Kyung-Hee Hong, Seung-Ah Yoo, Jin-Sun Kong, Jongseon Choe, Chul-Soo Cho

**Affiliations:** 1 Division of Rheumatology, Department of Internal Medicine, School of Medicine, Catholic University of Korea, Seoul 137-701, Korea; 2 Department of Microbiology and Immunology, Kangwon National University College of Medicine, Chunchon, Kangwon 200-701, Korea

## Abstract

The characteristics of rheumatoid arthritis (RA) pathology include the infiltration of inflammatory leukocytes, the proliferation of synovial cells, and the presence of extensive angiogenesis, referred to as rheumatoid pannus. Fas ligand is critical to the homeostatic regulation of the immune response, but its role in the angiogenic process of RA remains to be defined. In this study, we investigated whether soluble Fas ligand (sFasL) induces synoviocyte apoptosis and regulates angiogenesis of endothelial cells in RA. The levels of sFasL were elevated in the synovial fluids of RA patients when compared to those of osteoarthritis (OA) patients, and they correlated inversely with vascular endothelial growth factor_165 _(VEGF_165_) concentrations. sFasL, ranging from 10 to 100 ng/ml, induced the apoptosis of RA fibroblast-like synoviocytes (FLS) *in vitro*, and thereby decreased VEGF_165 _production. In addition, sFasL inhibited VEGF_165_-induced migration and chemotaxis of endothelial cells to basal levels in a manner independent of the Fas-mediated cell death. sFasL dose-dependently suppressed the VEGF_165_-stimulated increase in pAkt expression in endothelial cells, which might be associated with its anti-migratory effect on endothelial cells. Moreover, sFasL strongly inhibited neovascularization in the Matrigel plug *in vivo*. Our data suggest that sFasL shows anti-angiogenic activity within RA joints not only by inducing apoptosis of VEGF_165_-producing cells but also by blocking VEGF_165_-induced migration of endothelial cells, independent of Fas-mediated apoptosis.

## Introduction

Rheumatoid arthritis (RA) is a multi-systemic autoimmune disease of unknown etiology that is characterized by hyperplastic synovial membrane capable of destroying adjacent articular cartilage and bone [1,2]. The pathology of RA synovial membrane includes infiltration of inflammatory leukocytes, proliferation of synovial cells and extensive angiogenesis, which is collectively referred to as the rheumatoid pannus [2-4]. A critical phenomenon occurring in the early stages of synovial inflammation is angiogenesis [4,5], which commences with the activation of endothelial cells by a variety of stimuli, including pro-inflammatory cytokines and growth factors such as vascular endothelial growth factor (VEGF). The affected endothelial cells (ECs) then begin to digest the basement membrane, proliferate, migrate and eventually differentiate to form a tubular structure [6].

Fas (also known as CD95) was initially discovered as a cell surface molecule that efficiently triggers death signals when bound to its ligand, Fas ligand (FasL) [7,8]. Fas is ubiquitously expressed, whereas FasL is principally expressed on activated T cells [7], natural killer cells [9], tumor cells [10], and in immune privileged sites such as the eye [11]. The Fas-FasL interaction plays a pivotal role in activation-induced cell death of T lymphocytes [12], and is responsible for the cytotoxicity of T lymphocytes [13,14] and natural killer cells [9]. Consequently, Fas and FasL are crucial components of lymphocyte homeostasis. In addition to the homeostatic regulation of the immune system, Fas and FasL are involved in tumor surveillance [15,16]. Moreover, Fas and FasL are thought to inhibit angiogenesis by inducing apoptosis of either ECs or leukocytes that provide angiogenic growth factors [17-20], although one study reported an increase in angiogenesis by Fas and FasL [21]. Similar to tumor necrosis factor (TNF)-α, FasL is cleaved from the cell surface by a metalloproteinase [22]. The released form of FasL, soluble FasL (sFasL), was originally thought to induce apoptosis in a manner similar to membrane-associated FasL (mFasL) [23]. However, there have been many subsequent reports upholding the differences between sFasL and mFasL regarding apoptosis induction [24,25]. Despite the numerous studies on the role of Fas and FasL in immune homeostasis, the effect of sFasL on the angiogenic process of RA remains to be determined.

In this study, we tested whether sFasL can regulate angiogenesis and apoptosis of rheumatoid synoviocytes. We demonstrate here that sFasL potently decreased VEGF_165 _production by RA fibroblast-like synoviocytes (FLSs) by inducing apoptosis *in vitro*. In addition, sFasL effectively inhibited VEGF_165_-induced migration and chemotaxis of ECs, although it did not affect tube formation by ECs. The effect of sFasL on ECs was not due to Fas-mediated cell death, since sFasL did not change either spontaneous or VEGF_165_-stimulated EC proliferation or survival. Moreover, sFasL strongly inhibited neovascularization in the Matrigel plug *in vivo*. Taken together, sFasL inhibits angiogenesis within RA synovium not only by inducing apoptosis of VEGF_165_-producing cells such as FLSs, but also by blocking VEGF_165_-induced migration of ECs, independent of Fas-mediated apoptosis.

## Materials and methods

### Culture of RA synoviocytes and collection of synovial fluids

The RA FLSs were prepared from the synovial tissues of ten RA patients that were undergoing total joint replacement surgery, as described previously [26]. The mean age of the RA patients (9 females and 1 male) was 48.3 years. Eight patients had a positive rheumatoid factor. Osteoarthritis (OA) FLSs, isolated from 5 female OA patients (mean age 66.2 years), were used as a control. Synovial tissues were minced into 2 to 3 mm pieces, and treated for 4 hours with 4 mg/ml of type I collagenase (Worthington Biochemical, Freehold, NJ, USA) in DMEM at 37°C in a 5% CO_2 _atmosphere. Dissociated cells were then resuspended in DMEM, supplemented with 10% FCS, 2 mM glutamine, penicillin and streptomycin, and then plated in 75 cm^2 ^flasks. After overnight culturing, the non-adherent cells were removed and adherent cells cultivated in DMEM plus 10% FCS. Cultures were kept at 37°C in a 5% CO_2 _atmosphere, and the medium was replaced every 3 days. At confluence, the cells were passed by diluting 1:3 with fresh medium and re-cultured until used. Synoviocytes, from passages 4 through 8, were used for each experiment. The FLSs were washed in DMEM and then incubated for an additional 24 hours in serum-free DMEM supplemented with insulin-transferrin-selenium A (ITSA; Life Technologies, Grand Island, NY, USA). The cells (3 × 10^4 ^cells/well) were seeded in triplicate into 24-well plates (Nunc, Roskilde, Denmark) in serum-free DMEM (supplemented with ITSA) without or with transforming growth factor (TGF)-β (PeproTech, London, UK) in the presence of sFasL (10 to 100 ng/ml; MBL, Nagoya, Japan). After the indicated hours of incubation, cell viability was tested by 3-(4,5-dimethylthiazol-2-yl)-2,5-diphenyltetrazolium bromide (MTT) assay, and the cell-free media were collected to measure VEGF_165 _concentration in the culture supernatants. Synovial fluid was obtained with informed consent from RA and OA patients with joint effusion, and stored at -20°C in a refrigerator. All samples were obtained according to the guidelines approved by the Ethics Committee of the Catholic University of Korea.

### ELISA for sFas and VEGF_165_

The amount of sFasL and VEGF_165 _was measured by ELISA, as previously described [27]. Recombinant human sFasL and VEGF_165 _(R & D, Minneapolis, MN, USA) were used as a calibration standard. A standard curve was drawn by plotting the optical density versus the log of the concentration of sFas and VEGF_165_.

### Isolation and culture of endothelial cells

The ECs were isolated from normal-term umbilical cord vein by collagenase digestion, and then grown to confluence in 75 cm^2 ^flasks containing M199 medium (Life Technologies) supplemented with 20% FCS, 100 U/ml penicillin, 100 μg/ml streptomycin, and 2 mM L-glutamine. Cultures were kept at 37°C in a CO_2 _incubator, and the medium was changed every 2 to 3 days until confluence was reached. ECs were passed with 0.2% collagenase and 0.02% EDTA (Life Technologies); cells from passages 2 to 3 were used in this study.

### Determination of endothelial cell proliferation and viability

The proliferation and survival of ECs were assessed by [^3^H]-thymidine incorporation assay and MTT assay, respectively. Briefly, ECs were incubated for 24 hours in DMEM supplemented with 1% FCS. The medium was replaced with fresh DMEM/ITSA supplemented with 1% FCS, and the cells were incubated without or with 10 ng/ml of VEGF_165 _in the presence of sFasL (1 to 100 ng/ml) for 24 hours. For the determination of the proliferation rate of ECs, 1 μCi of [^3^H]-thymidine was added to each of the wells prior to the final 6 hours of culturing, and the incorporated radioactivity was counted with a scintillation counter.

### Wounding migration and tube formation assay

The wounding migration and tube formation activity of ECs were measured as described previously [28]. In brief, ECs plated at confluence on 60 mm culture dishes were wounded with pipette tips, and then treated with VEGF_165 _(20 ng/ml) in M199 medium, supplemented with 1% FCS and 1 mM of thymidine. After 12 hours of incubation, migration was quantified by counting the cells that had moved beyond the reference line. For the tube formation assay, ECs were seeded on a layer of previously polymerized Matrigel (BD Biosciences, San Jose, CA, USA) with VEGF_165 _(20 ng/ml). After 18 hours of incubation, the cell morphology was visualized via phase-contrast microscopy and photographed. The degree of tube formation was quantified by measuring the length of tubes in 5 randomly chosen low-power fields (×40) from each well using image-Pro Plus v4.5 (Media Cybernetics, San Diego, CA, USA).

### Chemotaxis assay of endothelial cells

The chemotactic migration of ECs was assayed using a Transwell chamber with 6.5 mm diameter polycarbonate filters (8 μm pore size). In brief, the filter's lower surface was coated with 10 μg of gelatin. VEGF_165 _(10 ng/ml), which was prepared in M199 medium containing 1% FCS, was placed in the lower wells. The ECs that were incubated in M199 with 1% FCS for 6 hours or overnight were trypsinized and suspended at a final concentration of 1 × 10^6 ^cells/ml in M199 containing 1% FCS. Various concentrations of sFasL or soluble CD40 ligand (sCD40L; R & D) were added to the upper wells with 100 μl of cell suspensions. The chamber was incubated at 37°C for 4 hours. The cells were fixed and stained with hematoxylin and eosin. Non-migrating cells on the filter's upper surface were removed by wiping with a cotton swab. Chemotaxis was quantified by counting the cells that migrated to the lower side of the filter by optical microscopy at ×200 magnification. Eight random fields were counted for each assay. Each sample was assayed in duplicate.

### Western blot analysis for phospho-Akt and phospho-ERK

ECs were incubated for 24 hours in DMEM with 1% FCS, and then VEGF_165 _(20 ng/ml) plus various concentrations of sFasL (1 to 50 ng/ml) were added to the cells for the indicated times. The treated ECs were then washed twice in PBS, dissolved in sample buffer (50 mM Tris-HCl, 100 mM NaCl, 0.1% SDS, 1% NP-40, 50 mM NaF, 1 mM Na_3_VO_4_, 1 μg/ml aprotinin, 1 μg/ml pepstatin, and 1 μg/ml leupeptin), boiled, separated via SDS-PAGE, and transferred to nitrocellulose membranes. After immunoblot analysis with anti-phospho-ERK1/2 (Thr202/Tyr204) or anti-phospho-Akt (Ser473), the membranes were stripped and re-incubated with β-actin antibody in order to detect total protein amounts.

### Mouse Matrigel plug assay

Matrigel (500 μl) containing VEGF_165 _(500 ng/ml) and heparin (9 U/ml) were injected subcutaneously with or without sFasL (100 ng/ml) into the abdomen of C57BL/6 mice (7 weeks of age), as described previously [28]. After 14 days, the skins of the mice were pulled back to expose the Matrigel plugs, which remained intact. After noting and photographing any quantitative differences, hemoglobin levels were measured by the Drabkin method, using a Drabkin reagent kit 525 (Sigma, St. Louis, MO, USA). The hemoglobin concentration was calculated from the parallel assay of a known amount of hemoglobin. The Matrigel plugs were fixed in 4% formalin, embedded with paraffin, and stained using hematoxylin and eosin.

### Statistical analysis

Data are expressed as the mean ± standard deviation (SD). Comparisons of the numerical data between groups were performed by paired or unpaired Mann-Whitney U-test. *P *values less than 0.05 are considered statistically significant.

## Results

### sFasL and VEGF_165 _levels correlate inversely in patients with RA

**Figure 1:**
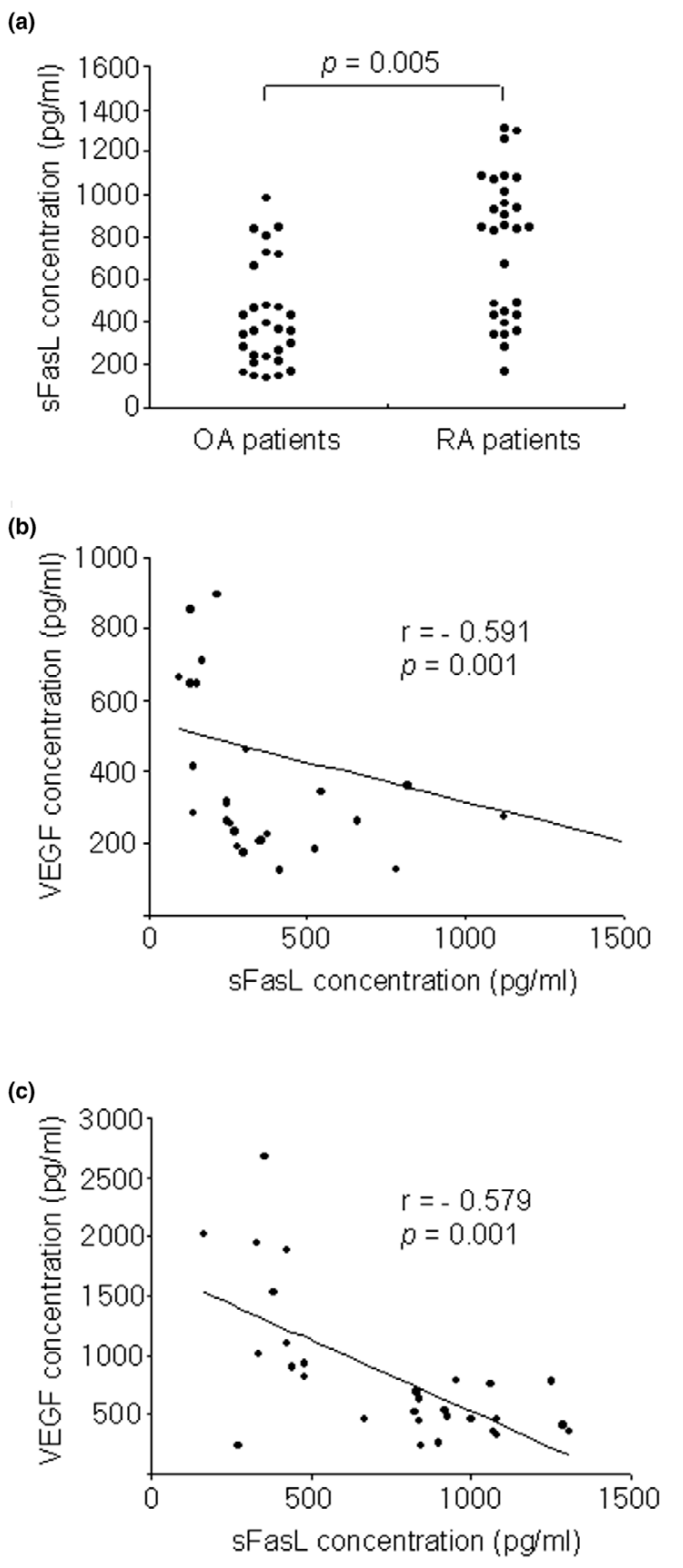
Inverse correlation between soluble Fas ligand (sFasL) and vascular endothelial growth factor (VEGF) levels in patients with rheumatoid arthritis (RA). **(a) **Concentrations of sFasL in synovial fluid of patients with RA (*n *= 29) and osteoarthritis (OA, *n *= 30). **(b) **Correlation of VEGF_165 _concentration with sFasL level in the sera of patients with RA. **(c) **Correlation between VEGF_165 _and sFasL levels in the synovial fluid of patients with RA.

Using ELISA, sFasL was detected in synovial fluids from RA patients (*n *= 29) and OA patients (*n *= 30) (Figure 1a). As with another reported study [27], the concentration of sFasL in the synovial fluid of RA patients was significantly higher than that of OA patients (mean levels of sFasL: 753 ± 335 versus 474 ± 401 pg/ml, *P *= 0.005). It has been reported that there is an inverse correlation between apoptotic (FasL, caspase-3) and angiogenic (VEGF, microvessel density) factors in squamous cell lung carcinomas [19]. To determine the relationship between sFasL and VEGF_165_, the serum and synovial fluids of RA patients were simultaneously measured for sFasL and VEGF_165 _by ELISA. As reported earlier [29], the VEGF_165 _level was significantly higher in synovial fluids of RA patients than those of OA patients (mean levels of VEGF_165_: 823 ± 615 versus 230 ± 131 pg/ml, *P *= 0.015). In RA patients, there were strong negative correlations between VEGF_165 _and sFasL levels in the sera (r = -0.591, *P *= 0.001) and synovial fluids (r = -0.579, *P *= 0.001) (Figure 1b,c). However, sFasL levels in OA synovial fluids did not show any correlation with VEGF_165 _concentrations (data not shown).

### sFasL decreases VEGF_165 _production by synovial fibroblasts via apoptosis induction

**Figure 2:**
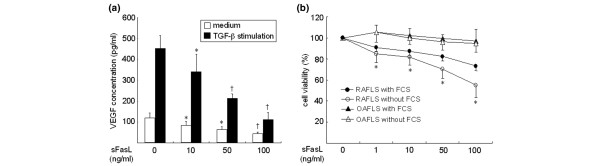
Effect of soluble Fas ligand (sFasL) on vascular endothelial growth factor (VEGF)_165 _production by synovial fibroblasts. **(a) **Fibroblast-like synoviocytes (FLSs) were cultured in triplicate for 24 hours with medium alone and transforming growth factor (TGF)-β (10 ng/ml) in the presence of various concentrations of sFasL (10 to 100 ng/ml). The amount of VEGF_165 _in the culture supernatants was determined by ELISA. Data are the mean ± standard deviation (SD) of three independent experiments in triplicate. *P < 0.05; ^†^*P *< 0.01 versus the cells stimulated with medium alone or TGF-β in the absence of sFasL. **(b) **The rheumatoid arthritis FLSs (RAFLS; *n *= 7) or osteoarthritis FLSs (OAFLS; *n *= 5) were treated with increasing concentrations of sFasL (1 to 100 ng/ml) in the absence or presence of 1% FCS for 24 hours. The viability of FLS was determined by the 3-(4,5-dimethylthiazol-2-yl)-2,5-diphenyltetrazolium bromide (MTT) assay. Data are expressed as the mean ± SD. *P < 0.01 versus OAFLS.

It is well known that FLSs are the major site of VEGF_165 _production in RA joints [30]. Based on the finding of an inverse correlation between sFasL and VEGF_165 _levels in RA synovial fluids, we attempted to determine the effects of sFasL on VEGF_165 _production by FLSs *in vitro*. For this, RA FLSs were cultured in the presence of sFasL for 24 hours either with medium alone or with the supplementary addition of TGF-β (10 ng/ml), since TGF-β has been described to strongly induce VEGF_165 _[31,32]. As seen in Figure 2a, sFasL, ranging from 10 to 100 ng/ml, dose-dependently decreased both spontaneous and TGF-β-stimulated VEGF_165 _production by RA FLSs cultured without FCS. The sFasL-induced decrease in VEGF_165 _production appears to be mediated by cell death, because similar concentrations of sFasL decreased FLS survival to 55% of the basal level, as determined by MTT assay (Figure 2b). The sFasL-mediated cell death was partially blocked by co-treating RA FLSs (*n *= 7) with 1% FCS (Figure 2b). OA FLSs (*n *= 5) were resistant to sFasL-induced cell death, indicating that the apoptotic action of sFasL may be specific to RA FLSs. These results, together with the data presented in Figure 1, suggest that sFasL may indirectly participate in anti-angiogenesis by eliminating VEGF_165_-producing cells in RA joints, but not in OA joints.

### Effect of sFasL on angiogenesis of ECs *in vitro*

**Figure 3:**
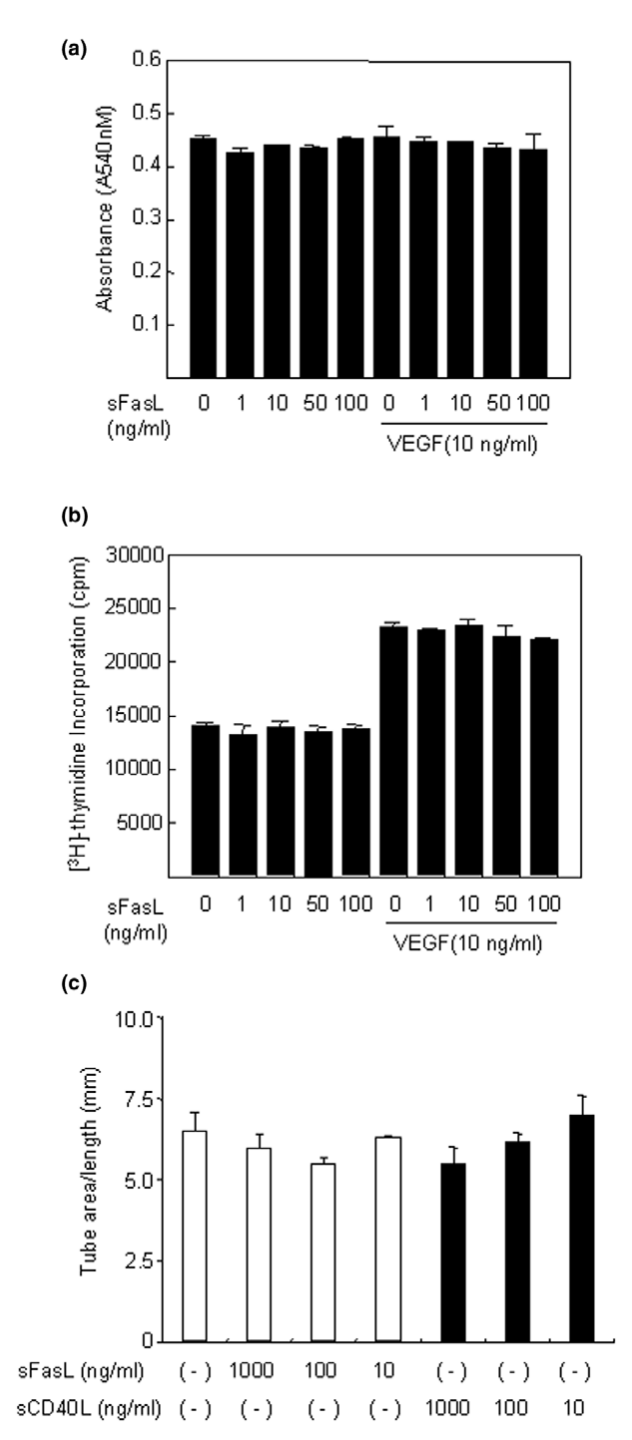
Soluble Fas ligand (sFasL) does not affect the survival, proliferation and tube formation of endothelial cells (ECs). **(a,b) **No effect of sFasL on the survival (a) and proliferation (b) of ECs. The ECs were incubated for 24 hours in DMEM supplemented with 1% FCS, and were treated with various concentrations of sFasL (1 to 100 ng/ml) in the absence or presence of vascular endothelial growth factor (VEGF)_165 _(10 ng/ml). The viability and proliferation of ECs were determined by the 3-(4,5-dimethylthiazol-2-yl)-2,5-diphenyltetrazolium bromide (MTT) and [^3^H]-thymidine incorporation assays, respectively. Data are expressed as the mean ± standard deviation (SD) of three independent experiments. **(c) **No effect of sFasL on tube formation by ECs. The ECs were plated on Matrigel matrices with VEGF_165 _(20 ng/ml) in the presence of various concentrations (10 to 1,000 ng/ml) of sFasL or soluble CD40 ligand (sCD40L) for 48 hours. The total length of the tube network was calculated using Image-Pro Plus software. Data are representative of three independent experiments with similar results, presented as the mean ± SD.

**Figure 4:**
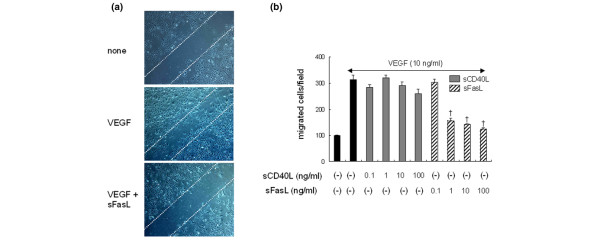
Soluble Fas ligand (sFasL) strongly inhibits migration and chemotaxis of endothelial cells (ECs). **(a) **Inhibition of wounding migration of ECs by sFasL. Confluent ECs were wounded with the tip of a micropipette, and incubated further in M199 containing 1% FCS with vascular endothelial growth factor (VEGF)_165 _(20 ng/ml) in the absence or presence of sFasL (50 ng/ml). After 12 hours, the cells migrating beyond the reference line were photographed (at ×50 magnification) and counted. A representative of three independent experiments is shown. **(b) **sFasL suppresses the chemotaxis of ECs induced by VEGF_165_. The ECs were placed in the upper wells of the chemotaxis chamber with various concentrations (0.1 to 100 ng/ml) of soluble CD40 ligand (sCD40L) or sFasL in the presence of VEGF (10 ng/ml) added in the lower wells. The number of cells that had migrated to the lower surface of the membrane was counted after hematoxylin and eosin staining. Data are representative of three independent experiments, presented as the mean ± standard deviation. ^†^*P *< 0.01 versus the VEGF_165_-stimulated cells without sFasL.

To investigate the effect of sFasL on the survival and proliferation of ECs, these cells were cultured with various concentrations of sFasL (1 to 100 ng/ml) in the absence or presence of 1% FCS. As expected, EC death was induced dose-dependently by treating the cells with sFasL in the absence of FCS (data not shown). However, when 1% FCS was added to the EC culture, sFasL failed to affect EC survival and proliferation, which were assessed by MTT assay and [^3^H]-thymidine incorporation assay, respectively (Figure 3a,b). The VEGF_165_-induced formation of tube-like structures by ECs was also not altered by the addition of sFasL (10 to 100 ng/ml; Figure 3c). In angiogenesis, ECs migrate in response to several chemotactic factors [6]. Therefore, we attempted to elucidate whether sFasL affects VEGF_165_-induced EC migration and chemotaxis. As shown in Figure 4a, sFasL suppressed VEGF_165_-induced wounding migration of ECs. Moreover, sFasL strongly inhibited VEGF_165_-induced chemotaxis of ECs, determined by using a Transwell chamber (Figure 4b), whereas sCD40L (0.1 to 100 ng/ml) did not affect it. These data suggest that sFasL inhibits angiogenesis by blocking migration and chemotaxis of ECs, independent of Fas-mediated cell death.

### sFasL inhibits VEGF_165_-induced upregulation of phospho-Akt expression

**Figure 5:**
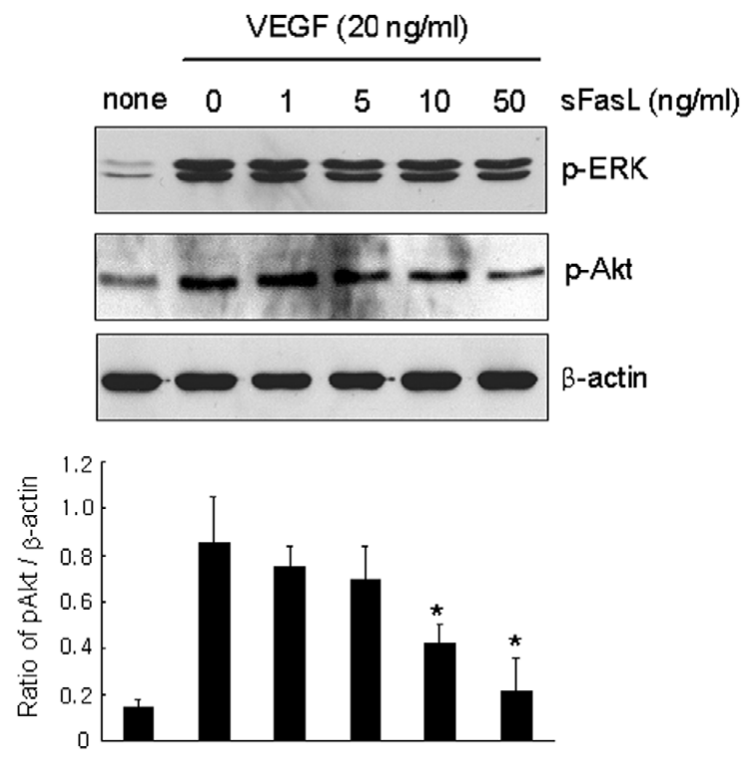
Inhibition of vascular endothelial growth factor (VEGF)_165_-induced phsospho-Akt (pAkt) activity by soluble Fas ligand (sFasL). Endothelial cells (ECs) were incubated with various concentrations of sFasL (1 to 50 ng/ml) in the presence of VEGF_165 _(20 ng/ml) for 10 minutes. The pAkt and phospho-extracellular signal-regulated kinase (pERK) expression levels in the ECs were determined by western blot analysis. The data are a representative result of three independent experiments, and are expressed as the mean (± standard deviation) optical density ratio [pAkt/β-actin]. *P < 0.05 versus the VEGF_165_-stimulated cells without sFasL.

VEGF_165 _exerts its biological effects by binding to its receptors, which include fms-like tyrosine kinase (Flt-1) and kinase insert domain-containing receptor (KDR) [33]. Flt-1 and KDR exhibit tyrosine kinase activity, and both are expressed in the majority of ECs [33,34]. KDR is a primary mediator of EC proliferation in response to VEGF_165_. Unlike KDR, Flt-1 is present in inflammatory cells, and mediates chemotaxis of ECs [33,34]. Thus, we sought to investigate whether sFasL regulates the expression of VEGF receptors or their signaling pathways in ECs. Our results show that sFasL failed to inhibit the mRNA and protein expression of Flt and KDR expression, as determined by reverse transcription PCR and Western blot analysis, respectively (data not shown). However, the VEGF_165_-induced expression of phospho-Akt (pAkt), one of the downstream targets for Flt signaling [35], was dose-dependently inhibited with sFasL treatment, whereas phospho-extracellular signal-regulated kinase (pERK) activity, which is critical for EC proliferation [35], remained unchanged (Figure 5). Taken together, these data suggest that sFasL may inhibit migration and chemotaxis of ECs by blocking VEGF_165_-induced upregulation of pAkt activity.

### sFasL inhibits angiogenesis *in vivo*

**Figure 6:**
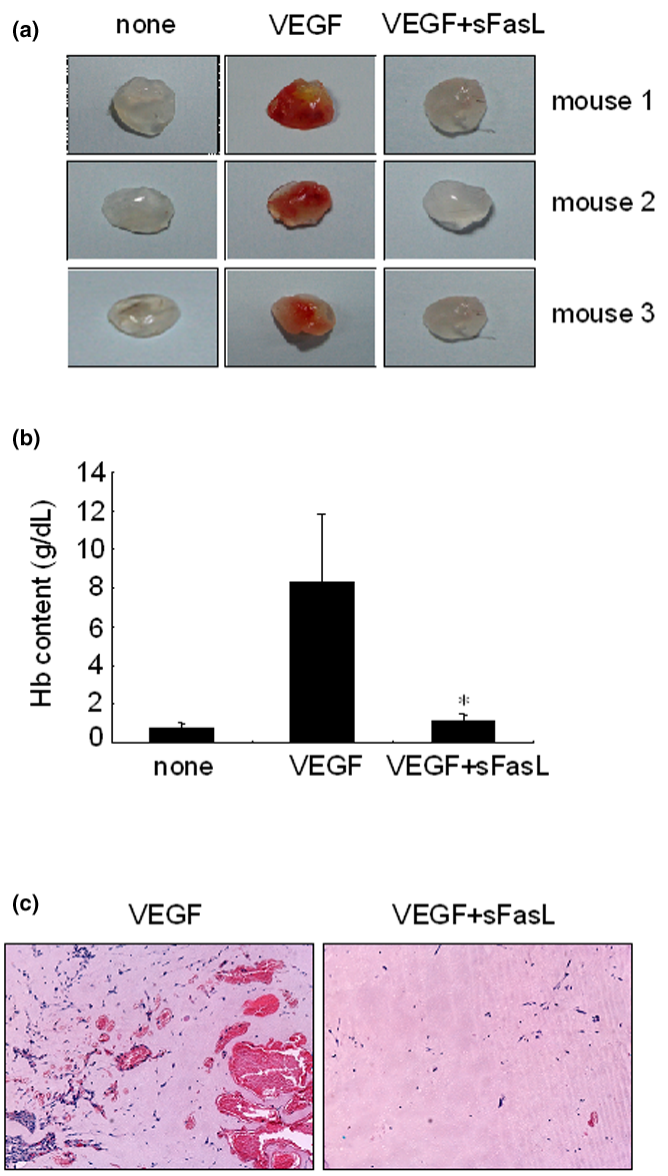
Effect of soluble Fas ligand (sFasL) on neovascularization *in vivo*. Matrigel containing vascular endothelial growth factor (VEGF)_165 _and heparin were injected subcutaneously with or without sFasL (100 ng/ml) into the abdomen of C57BL/6 mice. After 14 days, the mice were sacrificed and the Matrigel plugs were excised and fixed. **(a) **Representative Matrigel plugs containing PBS (none), VEGF_165 _(500 ng/ml), or VEGF_165 _(500 ng/ml) plus sFasL (100 ng/ml). **(b) **Quantification of new vessel formation via measurements of the hemoglobin (Hb) content within the Matrigel is shown. Eight mice were used for each group. Data represent the mean ± standard deviation, and similar results were obtained with two different experiments. *P < 0.001 versus the Hb content of the Matrigel containing VEGF_165_. **(c) **Representative photograph of the gels shown in cross-section and stained with hematoxylin and eosin. Original magnification (×100).

We finally attempted to determine whether sFasL blocks VEGF_165_-induced neovascularization *in vivo*. The *in vivo *exposed Matrigel mixtures containing VEGF_165 _(500 ng/ml) were colored orange to red, whereas the gels containing VEGF_165 _plus sFasL (100 ng/ml) retained their original white to amber coloring (Figure 6a). In an attempt to quantify the angiogenesis in these samples, we measured the hemoglobin contents of the Matrigel mixture gels. The mean hemoglobin content of the VEGF_165_-treated Matrigels (*n *= 8) was 8.30 ± 3.52 g/dl, whereas that of the VEGF_165 _plus sFasL-treated Matrigels (*n *= 8) was 1.20 ± 0.26 g/dl (*P *< 0.001) (Figure 6b). The stained sections indicate that Matrigels containing VEGF_165 _had produced more vessels in the gels than had the Matrigel containing the VEGF_165 _plus sFasL (Figure 6c). These new vessels were filled with an abundance of intact red blood cells, indicating the formation of a functional vasculature within the Matrigels. These results appear to suggest that sFasL has potent anti-angiogenic activity *in vivo *as well as *in vitro*.

## Discussion

FasL is a type II membrane protein of approximately 280 amino acids that belongs to the TNF/nerve growth factor family, which includes TNF, lymphotoxin, CD40L, and TRAIL. FasL induces apoptotic death in cells expressing its receptor, Fas [17,35]. FasL undergoes proteolytic cleavage in its extracellular domains, resulting in the release of sFasL [22]. In general, sFasL is less potent at inducing apoptosis than membrane-bound Fas, as shown in a variety of cell types [24,25,36]. Elevated levels of sFasL are found in sera from patients with atherosclerosis [37], leukemia [38], and acute graft-versus host disease [39]. The concentration of sFasL in the synovial fluid is higher in patients with severe RA than in those with mild RA or OA [27]. Moreover, local injection of sFasL into the affected joints suppresses experimental arthritis in rats [40], suggesting it has therapeutic potential in RA.

Angiogenesis has been considered a critical step in the progression of chronic arthritis, as well as an early determinant in the development of RA [41]. In this study, we first identified a novel mechanism for anti-angiogenesis in RA involving sFasL. sFasL decreased VEGF_165 _production from RA FLSs by inducing apoptosis *in vitro*. The apoptotic action of sFasL seems to be specific to RA FLSs because it minimally affected the viability of OA FLSs, which is consistent with the previous finding that OA FLSs are less sensitive to Fas-mediated apoptosis [42]. In addition, sFasL drastically suppressed VEGF_165_-induced migration and chemotaxis of ECs *in vitro *and also blocked neovascularization *in vivo*, although it did not alter the proliferation and tube formation of ECs, indicating that sFasL displays anti-angiogenic activity through at least two different mechanisms: induction of Fas-mediated cell death of VEGF_165_-producing cells; and apoptosis-independent inhibition of EC migration and chemotaxis. These data, together with earlier reports [15,40], suggest that administration of sFasL could be effective in treating several angiogenesis-dependent diseases, such as cancer and chronic inflammatory diseases, explaining how sFasL protects against the development of experimental arthritis.

RA FLSs are susceptible to anti-Fas IgM and undergo apoptosis *in vitro *[43,44]. By contrast, apoptotic cells are rarely observed in the RA synovium *in vivo *[43]. The effect of FasL on apoptosis of ECs is also still controversial; although some studies report that ECs are sensitive to FasL-induced apoptosis [45,46], others report the contrary [47,48]. In our experiments, sFasL was able to induce the apoptosis of RA FLSs and ECs in a culture condition without FCS (Figure 2b; data not shown). However, when 1% FCS was added to the medium, sFasL-mediated cell death was partially inhibited in the cultured FLSs or did not occur in ECs (Figures 2b and 3a), indicating that the apoptotic action of sFasL on VEGF-secreting cells, such as FLSs, was hampered by growth factors. Recently, we have demonstrated that VEGF_165 _protects FLSs from apoptotic death by regulating Bcl-2 expression and Bax translocation [49]. Therefore, it seems unlikely that sFasL greatly elicits apoptosis of FLSs and ECs in the joints with high levels of VEGF_165 _or other growth factors. Instead, a mechanism by which sFasL induces anti-angiogenesis by blocking migration and chemotaxis of ECs may be more relevant in this specific condition.

Fas ligation activates a pro-inflammatory program in some cell types independently of apoptotic responses [50-52]. For example, Fas stimulation increased expression of IκBα, matrix metalloproteinases and chemokines, and Fas-activated RA FLSs displayed increased chemotactic activity for monocytic cells [50]. In our study, Fas ligation by sFasL did not affect the production of chemokines, such as IL-8 and monocyte chemoattractant protein-1, by ECs (data not shown), suggesting that Fas ligation does not trigger chemotactic signals for ECs. On the contrary, sFasL treatment blocked the migration and chemotaxis of ECs induced by VEGF_165 _(Figure 4). Moreover, sFasL strongly inhibited VEGF_165_-induced pAkt activity, but not pERK activity, in ECs (Figure 5). It is unclear how sFasL regulates VEGF_165_-induced pAkt activity and then suppresses the chemotaxis and migration of ECs. Activation of the phosphatidylinositol 3-kinase/protein kinase Akt pathway mediates nitric oxide-induced EC migration and angiogenesis [53]. Conversely, inhibition of the Akt pathway results in anti-angiogenic effects through the inhibition of EC migration in an apoptosis-independent manner [54,55]. Given that the activities of Flt-1 and its downstream target pAkt are critical to VEGF_165 _signaling for chemotaxis [53,54], the anti-migratory action of sFasL may be mediated by blocking VEGF_165_-induced upregulation of pAkt activity. Further study is required to determine the molecular mechanisms for sFasL regulation of pAkt activity linked to chemotaxis control.

## Conclusion

We demonstrate here that FasL showed anti-angiogenic activity not only by inducing apoptosis of VEGF-producing cells but also by blocking VEGF-induced migration of ECs, independent of Fas-mediated apoptosis. Our data suggest that sFasL may be effective in the treatment of RA, and could be applicable to the modulation of various chronic VEGF-dependent inflammatory diseases.

## Abbreviations

CD40L = CD40 ligand; DMEM = Dulbecco's modified Eagle's medium; EC = endothelial cell; ELISA = enzyme-linked immunosorbent assay; FCS = fetal calf serum; FasL = Fas ligand; FLS = fibroblast-like synoviocyte; Flt = fms-like tyrosine kinase; ITSA = insulin-transferrin-selenium A; KDR = kinase insert domain-containing receptor; mFasL = membrane-associated FasL; OA = osteoarthritis; pAkt = phospho-Akt; PBS = phosphate-buffered saline; pERK = phospho-extracellular signal-regulated kinase; RA = rheumatoid arthritis; sFasL = soluble FasL; TGF = transforming growth factor; TNF = tumor necrosis factor; VEGF = vascular endothelial growth factor.

## Competing interests

The authors declare that they have no competing interests.

## Authors' contributions

WK participated in the design of the study, drafted the manuscript and analyzed the data. SK helped to draft the manuscript. KH carried out cell cultures and ELISA, and helped to perform migration, chemotaxis, and tube formation assays of ECs. JK and SU assisted in cell cultures and performed migration, chemotaxis, and tube formation assays of ECs, and the *in vivo *Matrigel assay. JC participated in the design of the study and the analysis of data. CC conceived of the study, participated in its design and coordination, and helped to draft the manuscript. All authors read and approved the final manuscript.

## References

[B1] Feldmann M, Brennan FM, Maini RN (1996). Rheumatoid arthritis. Cell.

[B2] Firestein GS (1996). Invasive fibroblast-like synoviocytes in rheumatoid arthritis. Passive responders or transformed aggressors?. Arthritis Rheum.

[B3] Reece RJ, Canete JD, Parsons WJ, Emery P, Veale DJ (1999). Distinct vascular patterns of early synovitis in psoriatic, reactive, and rheumatoid arthritis. Arthritis Rheum.

[B4] Koch AE (2003). Angiogenesis as a target in rheumatoid arthritis. Ann Rheum Dis.

[B5] Fearon U, Griosios K, Fraser A, Reece R, Emery P, Jones PF, Veale DJ (2003). Angiopoietins, growth factors, and vascular morphology in early arthritis. J Rheumatol.

[B6] Risau W (1997). Mechanisms of angiogenesis. Nature.

[B7] Suda T, Takahashi T, Golstein P, Nagata S (1993). Molecular cloning and expression of the Fas ligand, a novel member of the tumor necrosis factor family. Cell.

[B8] Owen-Schaub LB, Yonehara S, Crump WL, Grimm EA (1992). DNA fragmentation and cell death is selectively triggered in activated human lymphocytes by Fas antigen engagement. Cell Immunol.

[B9] Oshimi Y, Oda S, Honda Y, Nagata S, Miyazaki S (1996). Involvement of Fas ligand and Fas-mediated pathway in the cytotoxicity of human natural killer cells. J Immunol.

[B10] Strand S, Hofmann WJ, Hug H, Muller M, Otto G, Strand D, Mariani SM, Stremmel W, Krammer PH, Galle PR (1996). Lymphocyte apoptosis induced by CD95 (APO-1/Fas) ligand-expressing tumor cells – a mechanism of immune evasion?. Nat Med.

[B11] Griffith TS, Brunner T, Fletcher SM, Green DR, Ferguson TA (1995). Fas ligand-induced apoptosis as a mechanism of immune privilege. Science.

[B12] Ju ST, Panka DJ, Cui H, Ettinger R, el-Khatib M, Sherr DH, Stanger BZ, Marshak-Rothstein A (1995). Fas(CD95)/FasL interactions required for programmed cell death after T-cell activation. Nature.

[B13] Hanabuchi S, Koyanagi M, Kawasaki A, Shinohara N, Matsuzawa A, Nishimura Y, Kobayashi Y, Yonehara S, Yagita H, Okumura K (1994). Fas and its ligand in a general mechanism of T-cell-mediated cytotoxicity. Proc Natl Acad Sci USA.

[B14] Lowin B, Hahne M, Mattmann C, Tschopp J (1994). Cytolytic T-cell cytotoxicity is mediated through perforin and Fas lytic pathways. Nature.

[B15] Wajant H (2006). CD95L/FasL and TRAIL in tumour surveillance and cancer therapy. Cancer Treat Res.

[B16] Davidson WF, Giese T, Fredrickson TN (1998). Spontaneous development of plasmacytoid tumors in mice with defective Fas-Fas ligand interactions. J Exp Med.

[B17] Lee HO, Ferguson TA (2003). Biology of FasL. Cytokine Growth Factor Rev.

[B18] Kaplan HJ, Leibole MA, Tezel T, Ferguson TA (1999). Fas ligand (CD95 ligand) controls angiogenesis beneath the retina. Nat Med.

[B19] Volm M, Mattern J, Koomagi R (1999). Inverse correlation between apoptotic (Fas ligand, caspase-3) and angiogenic factors (VEGF, microvessel density) in squamous cell lung carcinomas. Anticancer Res.

[B20] Panka DJ, Mier JW (2003). Canstatin inhibits Akt activation and induces Fas-dependent apoptosis in endothelial cells. J Biol Chem.

[B21] Biancone L, Martino AD, Orlandi V, Conaldi PG, Toniolo A, Camussi G (1997). Development of inflammatory angiogenesis by local stimulation of Fas *in vivo*. J Exp Med.

[B22] Kayagaki N, Kawasaki A, Ebata T, Ohmoto H, Ikeda S, Inoue S, Yoshino K, Okumura K, Yagita H (1995). Metalloproteinase-mediated release of human Fas ligand. J Exp Med.

[B23] Tanaka M, Suda T, Takahashi T, Nagata S (1995). Expression of the functional soluble form of human fas ligand in activated lymphocytes. EMBO J.

[B24] Tanaka M, Itai T, Adachi M, Nagata S (1998). Downregulation of Fas ligand by shedding. Nat Med.

[B25] Schneider P, Holler N, Bodmer JL, Hahne M, Frei K, Fontana A, Tschopp J (1998). Conversion of membrane-bound Fas(CD95) ligand to its soluble form is associated with downregulation of its proapoptotic activity and loss of liver toxicity. J Exp Med.

[B26] Yoo SA, Park BH, Park GS, Koh HS, Lee MS, Ryu SH, Miyazawa K, Park SH, Cho CS, Kim WU (2006). Calcineurin is expressed and plays a critical role in inflammatory arthritis. J Immunol.

[B27] Hashimoto H, Tanaka M, Suda T, Tomita T, Hayashida K, Takeuchi E, Kaneko M, Takano H, Nagata S, Ochi T (1998). Soluble Fas ligand in the joints of patients with rheumatoid arthritis and osteoarthritis. Arthritis Rheum.

[B28] Lee MS, Moon EJ, Lee SW, Kim MS, Kim KW, Kim YJ (2001). Angiogenic activity of pyruvic acid in *in vivo *and *in vitro *angiogenesis models. Cancer Res.

[B29] Lee SS, Joo YS, Kim WU, Min DJ, Min JK, Park SH, Cho CS, Kim HY (2001). Vascular endothelial growth factor levels in the serum and synovial fluid of patients with rheumatoid arthritis. Clin Exp Rheumatol.

[B30] Fava RA, Olsen NJ, Spencer-Green G, Yeo KT, Yeo TK, Berse B, Jackman RW, Senger DR, Dvorak HF, Brown LF (1994). Vascular permeability factor/endothelial growth factor (VPF/VEGF): accumulation and expression in human synovial fluids and rheumatoid synovial tissue. J Exp Med.

[B31] Berse B, Hunt JA, Diegel RJ, Morganelli P, Yeo K, Brown F, Fava RA (1999). Hypoxia augments cytokine (transforming growth factor-beta (TGF-beta) and IL-1)-induced vascular endothelial growth factor secretion by human synovial fibroblasts. Clin Exp Immunol.

[B32] Pertovaara L, Kaipainen A, Mustonen T, Orpana A, Ferrara N, Saksela O, Alitalo K (1994). Vascular endothelial growth factor is induced in response to transforming growth factor-beta in fibroblastic and epithelial cells. J Biol Chem.

[B33] Ferrara N, Gerber HP, LeCouter J (2003). The biology of VEGF and its receptors. Nat med.

[B34] Autiero M, Luttun A, Tjwa M, Carmeliet P (2003). Placental growth factor and its receptor, vascular endothelial growth factor receptor-1: novel targets for stimulation of ischemic tissue revascularization and inhibition of angiogenic and inflammatory disorders. J Thromb Haemost.

[B35] Kowanetz M, Ferrara N (2006). Vascular endothelial growth factor signaling pathways: therapeutic perspective. Clin Cancer Res.

[B36] Sieg S, Smith D, Kaplan D (1999). Differential activity of soluble versus cellular Fas ligand: regulation by an accessory molecule. Cell Immunol.

[B37] Okura T, Watanabe S, Jiang Y, Nakamura M, Takata Y, Yang ZH, Kohara K, Kitami Y, Hiwada K (2002). Soluble Fas ligand and atherosclerosis in hypertensive patients. J Hypertens.

[B38] Saitoh T, Karasawa M, Sakuraya M, Norio N, Junko T, Shirakawa K, Matsushima T, Tsukamoto N, Nojima Y, Murakami H (2000). Improvement of extrathymic T cell type of large granular lymphocyte (LGL) leukemia by cyclosporin A: the serum level of Fas ligand is a marker of LGL leukemia activity. Eur J Haematol.

[B39] Kanda Y, Tanaka Y, Shirakawa K, Yatomi T, Nakamura N, Kami M, Saito T, Izutsu K, Asai T, Yuji K (1998). Increased soluble Fas-ligand in sera of bone marrow transplant recipients with acute graft-versus-host disease. Bone Marrow Transplant.

[B40] Li NL, Nie H, Yu QW, Zhang JY, Ma AL, Shen BH, Wang L, Bai J, Chen XH, Zhou T, Zhang DQ (2004). Role of soluble Fas ligand in autoimmune diseases. World J Gastroenterol.

[B41] Koch AE (1998). Review: angiogenesis: implications for rheumatoid arthritis. Arthritis Rheum.

[B42] Okamoto K, Fujisawa K, Hasunuma T, Kobata T, Sumida T, Nishioka K (1997). Selective activation of the JNK/AP-1 pathway in Fas-mediated apoptosis of rheumatoid arthritis synoviocytes. Arthritis Rheum.

[B43] Peng SL (2006). Fas (CD95)-related apoptosis and rheumatoid arthritis. Rheumatology (Oxford).

[B44] Baier A, Meineckel I, Gay S, Pap T (2003). Apoptosis in rheumatoid arthritis. Curr Opin Rheumatol.

[B45] Filippatos G, Ang E, Gidea C, Dincer E, Wang R, Uhal BD (2004). Fas induces apoptosis in human coronary artery endothelial cells *in vitro*. BMC Cell Biol.

[B46] Janin A, Deschaumes C, Daneshpouy M, Estaquier J, Micic-Polianski J, Rajagopalan-Levasseur P, Akarid K, Mounier N, Gluckman E, Socie G, Ameisen JC (2002). CD95 engagement induces disseminated endothelial cell apoptosis *in vivo*: immunopathologic implications. Blood.

[B47] Mogi M, Fukuo K, Yang J, Suhara T, Ogihara T (2001). Hypoxia stimulates release of the soluble form of fas ligand that inhibits endothelial cell apoptosis. Lab Invest.

[B48] Sata M, Suhara T, Walsh K (2000). Vascular endothelial cells and smooth muscle cells differ in expression of Fas and Fas ligand and in sensitivity to Fas ligand-induced cell death: implications for vascular disease and therapy. Arterioscler Thromb Vasc Biol.

[B49] Kim WU, Kang SS, Yoo SA, Hong KH, Bae DG, Lee MS, Hong SW, Chae CB, Cho CS (2006). Interaction of vascular endothelial growth factor _165 _with neuropilin-1 protects rheumatoid synoviocytes from apoptotic death by regulating bcl-2 expression and bax translocation. J Immunol.

[B50] Palao G, Santiago B, Galindo MA, Rullas JN, Alcami J, Ramirez JC, Pablos JL (2006). Fas activation of a proinflammatory program in rheumatoid synoviocytes and its regulation by FLIP and caspase 8 signaling. Arthritis Rheum.

[B51] Choi C, Xu X, Oh JW, Lee SJ, Gillespie GY, Park H, Jo H, Benveniste EN (2001). Fas-induced expression of chemokines in human glioma cells: involvement of extracellular signal-regulated kinase 1/2 and p38 mitogen-activated protein kinase. Cancer Res.

[B52] Ahn JH, Park SM, Cho HS, Lee MS, Yoon JB, Vilcek J, Lee TH (2001). Non-apoptotic signaling pathways activated by soluble Fas ligand in serum-starved human fibroblasts. Mitogen-activated protein kinases and NF-kappaB-dependent gene expression. J Biol Chem.

[B53] Kawasaki K, Smith RS, Hsieh CM, Sun J, Chao J, Liao JK (2003). Activation of the phosphatidylinositol 3-kinase/protein kinase Akt pathway mediates nitric oxide-induced endothelial cell migration and angiogenesis. Mol Cell Biol.

[B54] Dell'Eva R, Ambrosini C, Minghelli S, Noonan DM, Albini A, Ferrari N (2007). The Akt inhibitor deguelin, is an angiopreventive agent also acting on the NF-κB pathway. Carcinogenesis.

[B55] Murtagh J, Lu H, Schwartz EL (2006). Taxotere-induced inhibition of human endothelial cell migration is a result of heat shock protein 90 degradation. Cancer Res.

